# Trends of Stem Cell Therapies in Age-Related Macular Degeneration

**DOI:** 10.3390/jcm10081785

**Published:** 2021-04-20

**Authors:** Tadao Maeda, Sunao Sugita, Yasuo Kurimoto, Masayo Takahashi

**Affiliations:** 1 Department of Ophthalmology, Kobe City Eye Hospital, Kobe 650-0047, Japan; tadao_maeda@kcho.jp (T.M.); sunao.sugita@riken.jp (S.S.); ykurimoto@mac.com (Y.K.); 2 Laboratory for Retinal Regeneration, RIKEN Center for Biosystems Dynamics Research, Kobe 650-0047, Japan

**Keywords:** regenerative medicine, retinal pigment epithelium, iPS cell, ES cell, stem cell, age-related macular degeneration, clinical trial, retina, immune reaction, transplantation

## Abstract

Age-related macular degeneration (AMD) is a highly prevalent irreversible impairment in the elderly population worldwide. Stem cell therapies have been considered potentially viable for treating AMD through the direct replacement of degenerated cells or secretion of trophic factors that facilitate the survival of existing cells. Among them, the safety of pluripotent stem cell-derived retinal pigment epithelial (RPE) cell transplantation against AMD, and some hereditary retinal degenerative diseases, has been discussed to a certain extent in clinical studies of RPE cell transplantation. Preparations are in progress for its clinical application. On the other hand, clinical trials using somatic stem cells are also being conducted, though these had controversial outcomes. Retinal regenerative medicine using stem cells is expected to make steady progress toward practical use while new technologies are incorporated from various fields, thereby making the role of ophthalmologists in this field increasingly important.

## 1. Introduction

Age-related macular degeneration (AMD) is one of the most common causes of blindness worldwide, especially in the elderly population. As the global prevalence is 8.7% and the age of onset varying from 45 to 86 years, it is estimated to affect approximately 288 million individuals in western countries by 2040 [1]. Given the diverse variations among ethnicities, AMD is 10 times more prevalent among Caucasians compared to African-Americans. The early stages of AMD are characterized by the hallmarks, known as drusen and depigmentation of the retinal pigment epithelium (RPE) cells. Its progression from early to intermediate and advanced levels is driven by the increase in the numbers of drusen and degenerated RPE cells, resulting in pigmentary changes and the formation of choroidal neovascularization (CNV). The advanced stages of AMD are categorized into two forms: Non-neovascular (dry, non-exudative, or geographic) and neovascular (wet or exudative). Dry AMD is characterized by geographic atrophy of the RPE, photoreceptor, and choriocapillaris, resulting in gradual retinal cell loss and decreased visual acuity. In the wet-type AMD, CNV causes sub-retinal leakage of blood, lipids, fluids, and the formation of fibrous scars. Currently, AMD patients are recommended to receive routine medical management, including antioxidant supplements and anti-vascular endothelial growth factor (anti-VEGF) agents. The former, including vitamins, lutein, and zeaxanthin, are applied to protect the retinal cells from oxidative stress. Meanwhile, intravitreal injection of anti-VEGF agents, such as ranibizumab, aflibercept, and bevacizumab, which bind to VEGF receptors to block VEGF, is commonly used for treating wet-type AMD. However, current treatments do not target the underlying degeneration inherent in the disease, leading to a high recurrence rate upon the discontinuation of treatment. Furthermore, there are currently no effective methods for treating dry-type AMD. To address these problems, retinal cell therapy has attracted worldwide attention as the new era of treatment for retinal degenerative diseases [2,3,4,5], such as reconstruction and functional recovery of RPE by cell transplantation to maintain or restore visual function.

Currently, there are two types of formulations used for the administration of RPE cell products, namely, cell sheets with or without scaffolds and cell suspensions. In the case of RPE cell sheet transplantation, various dedicated devices have been used in previous publications [2,6,7,8]. Meanwhile, a soft-tip sub-retinal cannula is used for transplanting an RPE cell suspension [2,9,10,11]. Generally, the risk of surgical complications of RPE sheet transplantation is higher than RPE cell suspension due to the greater surgical invasiveness, involving a wider incision site and occasional removal of CNV before RPE sheet transplantation. The safety results of the transplantation of pluripotent stem cell-derived retinal pigment epithelial cells (RPE) in both formulations have been described in previous literature [6,7,8,9,10,11,12,13,14,15]. Here, we describe the current status and prospects of retinal regenerative medicine for AMD using pluripotent stem cell-derived RPE and somatic stem cells.

The conceptual mode of action of pluripotent stem cell-derived RPE cells for wet-type AMD (A) and dry-type AMD (B) in either formulation, RPE cell sheet, or RPE cell suspension were shown, respectively.

## 2. History of RPE Cell Therapy for Age-Related Macular Degeneration

Research on RPE cell transplantation began attracting attention in the late 1980s. Transplanting human RPE cells into a monkeys’ sub-retinal space revealed engraftment on Bruch’s membrane [16]. Since then, several reports have been published on the protective effect of RPE cell transplantation on the neural retina in animal models [2], demonstrating the possibility of securing materials for photoreceptor cells and RPE for use in the cell therapy of diseases with impaired retinal outer layer. Additionally, a proof of concept for treatment was obtained for the RPE.

In humans, Peyman first reported RPE transplantation in patients with AMD in 1991 [17]. In the first case, autologous cell transplantation was performed after removing the proliferative tissue under the macula. The nearby RPE was then transplanted into the macula to improve visual acuity. In the second case, the RPE was exfoliated from the donor’s eye as a sheet before being transplanted, but no visual acuity improvement was observed. The AMD-related CNV was removed, and a cell sheet obtained by culturing fetal-derived RPE was transplanted [18,19], but immune rejection occurred after the operation. Weisz also attempted injecting the fetal RPE as a cell suspension, but no improvement in the visual acuity was observed. Graft fibrosis was also observed [20]. Meanwhile, Del Priore transplanted a donor RPE sheet after removing the CNV, but the poor engraftment and visual acuity did not improve [21]. Almost all transplants using allografts in the eyes with a damaged blood-retinal barrier due to CNV removal showed rejection and deterioration in visual acuity.

Autologous transplantation is ideal for avoiding rejection. For some time, the RPE used for transplant was frequently collected from the peripheral area [22,23,24]. Although some patients had improved visual acuity, it was difficult to collect a sufficient number of autologous RPE cells with stable quality, and serious adverse events frequently occurred due to surgical invasion. In addition, among patients transplanted with peripheral RPE patches with the choroid, some resulted in improved visual acuity, but the surgical procedure had a higher risk of lacerating the patches. Furthermore, the choroid acted as a fibrous tissue if it was not connected to the host choroidal vessels.

As a countermeasure to these problems, the transplanted cell source was reviewed, and we reported RPE cells derived from pluripotent stem cells (ES cells, iPS cells) as candidate graft cells [25,26,27]. RPE cells derived from ES and iPS cells have the same functions as those derived from living organisms, and these cells form cell sheets through the collection of elegant hexagonal cells with tight junctions. Due to their easier preparation compared to primary cultured RPE cells, these have made dramatic developments in the cell therapy for AMD. Furthermore, RPE cell transplantation advanced first among the pluripotent stem cells due to the following characteristics of ES/iPS cell-derived RPE cells, making them more suitable for clinical application: (1) They have the required functions (quality), (2) the retina requires a small number of cells so that enough can be manufactured for transplantation (amount), (3) cells with certified quality for clinical use can always be obtained (reproducibility), and (4) the standard of purity was satisfied because of the color (purity). Furthermore, sub-retinal surgeries, such as CNV removal, have already been performed in the past. Thus, as described above, the field of ophthalmology has contributed greatly to the clinical application of pluripotent stem cells.

## 3. Cell Therapy for Age-Related Macular Degeneration Using Pluripotent Stem Cell-Derived RPE Cells

**Figure 1:**
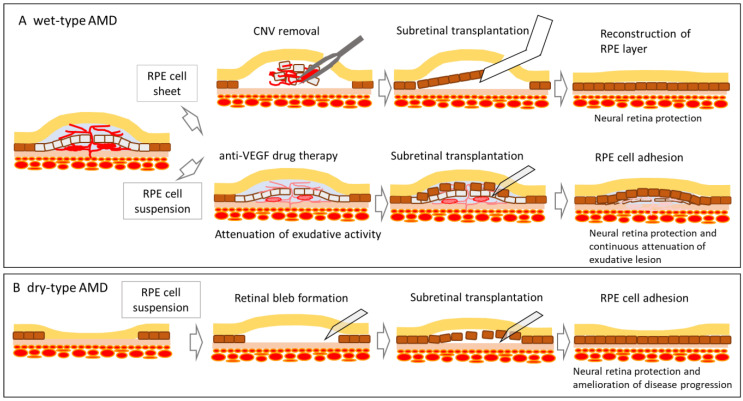
A conceptual mode of action of cell therapy using pluripotent stem cell-derived RPE cell products.

This section describes the implementation status of clinical studies on pluripotent stem cells in terms of the raw materials and the dosage form of the final product. The mode of action of cell therapy using pluripotent stem cell-derived RPE cells is shown in Figure 1, and a summary of clinical trials using pluripotent stem cell-derived RPE cells, with an updated status as of 31 December 2020 is presented in Table 1.

### 3.1. Autologous iPS Cell-Derived RPE Cell Sheet Transplantation

**Figure 2:**
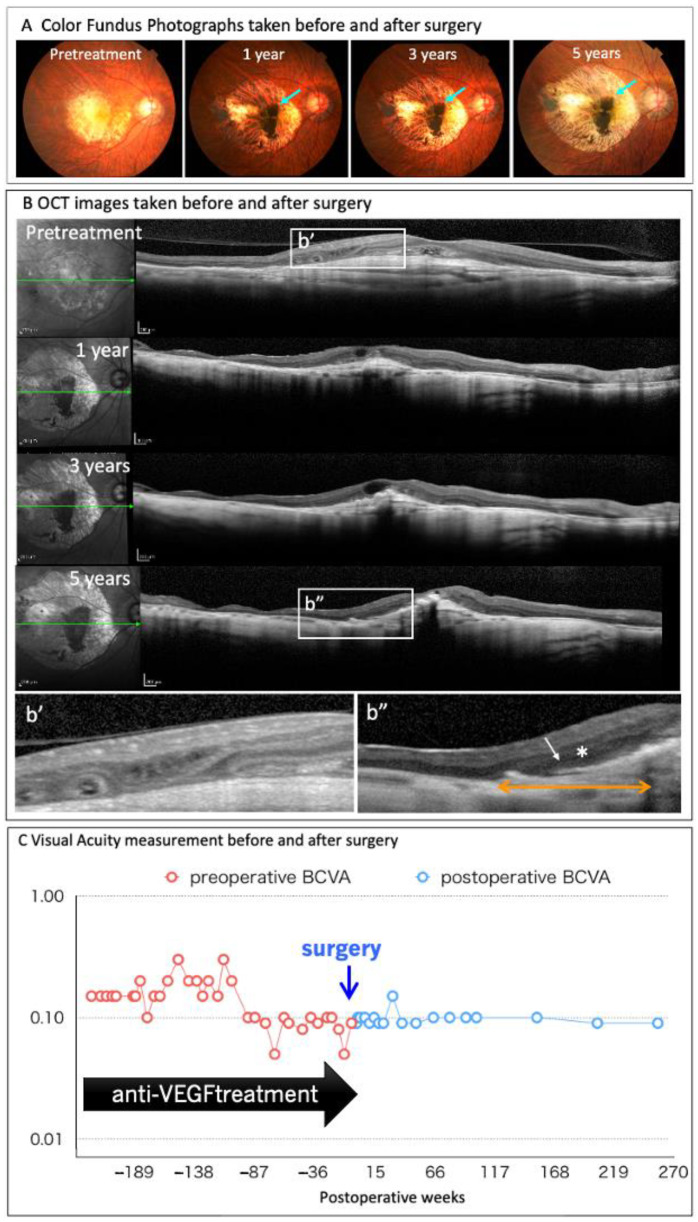
Clinical findings of the autologous iPSC-derived RPE sheet transplanted to wet-type AMD patient at five years after surgery.

In August 2013, Takahashi launched a joint clinical research on autologous iPS cell-derived RPE cell sheet transplantation with RIKEN, Institute of Biomedical Research and Innovation Hospital, and Kobe City Medical Center General Hospital (UMIN000011929). Each subject’s skin was collected in November 2013, and CNV removal and RPE sheet transplant surgery were performed in September 2014. In September 2015, 12 months after the transplant surgery, safety and effectiveness were evaluated as the primary and secondary endpoints, respectively [6]. The four-year report has already been published [12], and the five-year follow-up has also been completed (Figure 2).

At five years after the surgery, the autologous iPSC-derived RPE sheet is stable the in sub-retinal transplantation site, while maintaining pigmentation. A slight increase in graft size is also noted (A, green arrows). Changes in the retinal structure before, and after, surgery are monitored using optical coherence tomography (OCT) (B). The ONL layer (*) and IS/OS line are identified at the RPE sheet-transplanted lesion (above the orange arrow), whereas the retinal structure is disrupted before surgery (b’). The patient’s corrected visual acuity is maintained after transplantation for five years without significant changes, and the anti-VEGF antibody treatment was completely discontinued after surgery (C).

None of the primary endpoints, including intraoperative complications, tumorigenesis, engraftment failure, rejection, or other severe complications of transplanted cells, were observed five years after surgery. Furthermore, CNV recurrence was not observed without additional anti-VEGF antibody treatment. The corrected visual acuity was maintained at 0.09, preoperatively. Regarding the graft, the area of the graft was 1.521 mm^2^ at three days after transplantation, increasing three-fold to 5.471 mm^2^ at one year after transplantation. The size of the RPE sheet was relatively stable three years after transplantation. The choroidal volume below the transplanted graft was relatively stable at five years after transplantation. Whereas, the volume of the choroid outside of the lesion covered with the graft decreased to 60% compared to the pre-transplantation volume. In addition, the National Eye Institute is conducting research to evaluate the safety and feasibility of subretinal transplantation using iPSC-derived RPE grown as a monolayer on a biodegradable poly lactic-co-glycolic acid scaffold, as a potential autologous cell-based therapy for GA preparations associated with AMD.

### 3.2. Allogeneic iPS Cell-Derived RPE Cell Suspension Transplantation

**Figure 3:**
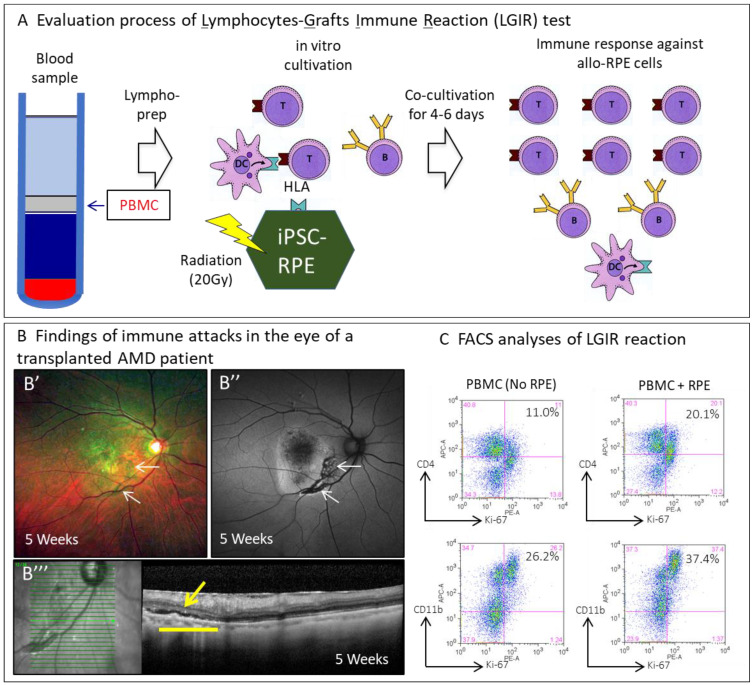
Evaluation of immune reaction against allogeneic iPSC-derived RPE cell transplantation with lymphocytes-grafts immune reaction (LGIR) test.

It was the first autologous iPS cell-derived retinal cell transplantation and was monitored worldwide [28], and its results suggest that this procedure is safe and efficacious. The next step was allogeneic transplantation. First, we established a test system called lymphocyte-graft cell immune reaction (LGIR) tests to evaluate and manage immune rejection for allogeneic RPE transplantation (Figure 3). We also established a test to detect grafted RPE-specific antibodies (donor-specific antibodies) [9].

The evaluation process of the LGIR test is presented (A). PBMCs are prepared by lympho-prep from a blood sample of the patient and then cultivated together with the iPSC-derived RPE cells comparable to those for transplantation. After co-cultivation for four-to-six days, the proliferation of immune cells against iPSC-derived RPE cells is evaluated by flow cytometry analysis. Findings of immune attacks in the eyes of a patient with transplanted AMD (B). Color fundus photographs at five weeks after surgery of the transplanted AMD patient (B’), and the autofluorescence fundus photographs (B’) and transplanted cells are observed (white arrows). Optical coherence tomography (OCT) image, grafted area (yellow bar) at five weeks (B’’). There are small amounts of sub-retinal fluid in the sub-retinal space (yellow arrow). The secretion pattern of inflammatory cytokines and cell proliferation of PBMCs are monitored by flow cytometry analysis (C). The iPS cell-derived RPE (iPSC-RPE) is treated with 20 Gy radiation to prevent proliferation during the assay. T: T cell, B: B cell, DC: dendritic cell, PBMC: peripheral blood mononuclear cells

In our preclinical studies [29,30,31], we confirmed the reliability of LGIR tests in detecting immune rejection after RPE cell transplantation. A local steroid administration protocol, a clinical study of allogeneic iPS cell-derived RPE suspension transplantation (UMIN000026003), was conducted in collaboration with RIKEN, Center for iPS Cell Research and Application, Kyoto University, Osaka University, and Kobe City Medical Center General Hospital. This study aimed to investigate the safety of six-loci HLA-matched allogeneic cell transplantation under local steroids only in order to standardize future cell therapies with iPS cell-derived RPE [9]. From March 2017 to September 2017, transplantation was performed on five patients with exudative AMD who had the same HLA haplotype identity as an allogeneic iPS-cell-derived RPE. The safety and efficacy were evaluated as primary and secondary endpoints, respectively, at 12 months after the transplantations. The following are notable points in allogeneic RPE transplantation:

1. The raw material was established from an HLA 6-locus homozygote donor manufactured by the Center for iPS Cell Research and Application, Kyoto University.
2. A cell suspension that is easy to store and transport and is considered less invasive by transplantation was selected as the dosage form.
3. A frozen stock of RPE cells was created as an intermediate. This was then thawed k based on the patient’s date of transplantation. A recovery culture was performed for two weeks, after which the cell suspension was prepared using a dedicated transplant medium.
4. At the time of transplantation, removal of the CNV, performed in autologous RPE transplantation was not conducted, and a commercially available ophthalmic cannula (PolyTip^®^ cannula 25 g/38 g, MedOne, FL, USA) was used as the dedicated transplantation device for the cell suspension.
5. Immune rejection after transplantation was evaluated using the LGIR and donor-specific antibodies tests to detect the response of the transplanted patients’ peripheral lymphocytes to the transplanted cells in vitro and the presence of RPE-specific antibodies. Optical coherence tomography (OCT) imaging was utilized to detect exudative findings in RPE-transplanted lesions.

There were no intraoperative complications, and the RPE cell suspension was implanted sub-retinal as planned in all five cases. According to the protocol, anti-VEGF antibody treatment and topical ocular steroids should be administered at the time of transplantation to treat the underlying disease and suppress rejection. Five weeks after transplantation, only one out of the five cases was suspected of having mild immune rejection from the OCT findings. The patient’s LGIR was slightly positive, indicating subtle immune rejection. As such, an additional sub-Tenon topical steroid was administered, resulting in successful management. No obvious rejection of the transplanted cells was observed during the observation period. The rejection reaction can, thus, be managed with topical ocular steroids. In addition, of the three cases with a positive LGIR, only one presented with clinical findings suggestive of rejection of transplanted cells. Furthermore, none presented with damage to the photoreceptor cells directly above the transplanted cells. In addition, the epi-retinal membrane was observed in all cases, and macular edema resistant to anti-VEGF antibody treatment was observed in only one case and was relieved through vitreous surgery. In the end, grafted RPE cells survived in all five cases for more than two years.

These study’s observations suggest that it is possible to manage immune rejection and complications appropriately.

### 3.3. Allogeneic ES Cell-Derived RPE Cell Suspension Transplantation

Ocata Therapeutics (formerly known as Advanced Cell Technology), a US venture company, submitted a clinical trial to the FDA in November 2009 for the transplantation of human ES cell-derived RPE cells for Stargardt’s disease (SMD), a hereditary macular degenerative disease, which was approved in November 2010 (NCT01345006). In January 2011, a clinical trial targeting atrophic AMD was approved (NCT01344993), and in July 2011, 250,000 RPE cells were transplanted to each patient [9]. In addition, the company has begun clinical trials for SMD in the United Kingdom [13]. Since ES cell-derived RPE cell transplantation is an allograft transplantation, this clinical trial was combined with immunosuppressive drug administration for three months. In October 2014, they reported the results of transplantation in nine cases each of SMD and atrophic AMD, revealing an increased pigmentation in 13 of the 18 cases. This effect was thought to be due to the proliferation of RPE cells at the transplant site. However, one patient developed post-surgery bacterial endophthalmitis after discontinuing the intake of immunosuppressive drugs. This was relieved after two months with the infusion of antibiotics and eye drops. The causative organism was not detected in the transplanted cells. Other reported systemic adverse events were due to the combined use of immunosuppressive drugs [19,20]. It has been reported that the administration of 50,000 to 200,000 RPE cells was completed in March 2015, leading to a total of 38 SMD and AMD subjects [10].

Similarly, in November 2011 at Moorfield Eye Hospital in the United Kingdom, 50,000 to 200,000 ES cell-derived RPE cell transplants were performed in 12 SMD cases, administered with immunosuppressive drugs for 13 weeks after transplantation [13]. In November 2018, the results of transplantation in the same 12 cases were reported (NCT01469832, NCT02941991). Twelve months after transplantation, no abnormal proliferation or inflammatory reaction of the transplanted cells was observed. In addition, while pigmentation increased at the RPE cell transplantation site, significant improvements in visual function, including visual acuity, retinal sensitivity, and QOL evaluation, were not observed. In the high-dose group, the retinal sensitivity decreased at the transplant site, where an increase in pigmentation was observed. In contrast, the retinal sensitivity tended to improve at the site where the increase in pigmentation was not observed. Table 1 presents the details of ongoing clinical trials in other institutions.

### 3.4. Allogeneic ES Cell-Derived RPE Cell Sheet

Two research groups recently published the results of their clinical trials. In a phase I clinical trial started in June 2015 at the Moorfield Eye Hospital, University of London, UK, two patients with exudative AMD with severe subretinal hemorrhage, measuring 6.0 mm long and 3.0 mm wide (area of 17.0 mm^2^), underwent RPE sheet transplantation using approximately 100,000 ES cell-derived RPE cells on a polyester scaffold material with a vitronectin-coated surface. Based on the results reported in March 2018 (NCT01691261) [7], no adverse events occurred, suggesting a clear causal relationship with the transplanted cells occurred during the one-year observation period. However, one case experienced retinal detachment and a ruptured lens sac after transplantation. It should be noted that the oral administration of steroids resulted in no obvious immune rejection. In contrast, immunosuppressive drugs, such as tacrolimus, were administered after transplantation in the above-mentioned clinical trial conducted by Ocata [5,10,11]. Although the primary endpoints of safety and improved visual acuity were confirmed in these two cases, it is possible that the visual acuity improved due to the lavage of the sub-retinal hemorrhage, making the involvement of cell transplantation in improving visual acuity unclear. On the other hand, at the Roski Eye Institute of The University of Southern California in the United States, four of the five cases enrolled in a Phase I/IIa clinical trial started in October 2015 had atrophic AMD measuring 6.25 mm long and 3.5 mm wide [8]. An RPE sheet was transplanted using approximately 100,000 ES cell-derived RPE cells on a scaffolding material made of porous parylene (area of 22.0 mm^2^), and the results were reported in April 2018 [8]. In this study, tacrolimus, an immunosuppressant, was administered for 60 days after transplantation. Similar to the sheet transplantation in the aforementioned UK study, there were no adverse events correlated with the transplanted cells, but severe sub-retinal hemorrhage occurred in one case, requiring additional treatment. At the time of reporting, the observation period was 120 to 365 days, with improved visual acuity in one case and improved fixation in three cases. In addition, the formation of the anterior retinal membrane was observed in patients with transplanted eyes. Table 1 presents the details of ongoing clinical trials in other institutions. Due to recent breakthroughs in scaffold formation, fully biodegradable scaffolds made of fibrin hydrogel [32,33] or poly lactic-co-glycolic acid have attracted attention as advanced alternatives for RPE cell sheet transplantation.

### 3.5. Possibility of Pluripotent Stem Cell-Derived RPE Cell/Photoreceptor Complex Product

As mentioned above, cell therapy with pluripotent stem cell-derived RPE cells for AMD is in progress. However, there is no established treatment for end-stage cases with absent photoreceptor cells. Our group transplanted retinal tissue differentiated from mouse and human ES cells and iPS cells into a mouse and rat with end-stage retinal degeneration using the method of retinal organoid formation from ES cells developed by Eiarku et al. [34]. We confirmed the proof of concept using the following steps: (1) The differentiated retinal sheets induced from ES/iPS cells via these culture systems were engrafted, resulting in maturation, while forming the entire structure of photoreceptor cells and maintaining reproducibility in all animal models [34,35,36,37]; (2) we also demonstrated the possibility that photoreceptor cells in transplanted tissues form synapses with host bipolar cells using genetic labeling of synaptic markers and immunostaining; (3) functionally, after transplantation, the retinal ganglion cells of the host mouse and rat retina provide a photoresponse and photoreaction that is not seen in the untransplanted retina; (4) in addition, among blind mice with degenerated retina (*rd1-/-*) transplanted with mouse iPS cell-derived retinal sheets, a behavior test revealed that about 40% sensed the light after transplantation. These observations verified the effectiveness of the sheet in clinical practice.

Furthermore, a proof of concept study using a non-human primate retinal degeneration model revealed that both ES cell- and iPS cell-derived retinal sheets mature in the sub-retinal space in mouse and rat models after transplantation. Furthermore, the study confirmed the long-term survival of the transplanted sheet (more than 2 years) and recovery of visual function at the same transplant site. Given these results, in October 2020, our group conducted the first clinical study using hiPS cell-derived retinal tissue for patients with retinitis pigmentosa. In the future, it is expected that the visual function can be recovered by performing simultaneous transplantation using a composite product of RPE cells and photoreceptor cells for advanced cases in which photoreceptor cells are lost.

### 3.6. Treatment with Somatic Stem Cells

Somatic stem cells were also tested in clinical trials to determine if these protected and improved the retinal circulation of the humoral factors secreted from transplanted cells instead of cell therapy based on retinal tissue reconstruction. We have summarized clinical trials using somatic stem cells with updated status by the end of December 2020 (Table 2). Most utilized bone marrow stem cell transplantation but varied in the route of administration, including intrathecal, subtenon sac, and retrobulbar injection. The target diseases include AMD, retinitis pigmentosa, and ischemic retinal disease. In addition, sub-retinal transplantation of neural stem cells for AMD has also been performed, but the results are unknown (Table 2). Unfortunately, severe cases of visual loss after injection have been reported, which are associated with ocular hypertension, hemorrhagic retinopathy, vitreous hemorrhage, combined traction and rhegmatogenous retinal detachment, or lens dislocation after intravitreous transplantation of adipose tissue-derived stem cells [38].

## 4. Future Prospective in Retinal Regenerative Medicine

### 4.1. Expanding Indications for RPE Cell Transplantation

While the RPE is responsible for maintaining retinal homeostasis [39,40,41], it has been suggested that its functional deterioration may be the primary cause of some hereditary and non-hereditary retinal degenerative diseases, such as AMD [40,42,43]. The causes of RPE impairment are both hereditary and non-hereditary, but the pathophysiology of retinal degeneration caused by RPE impairment is common. Therefore, these retinal degenerative diseases are grouped into a group of diseases called “RPE impaired disease,” regardless of whether they are hereditary or not; pluripotent stem cell-derived RPE cells can be used for these diseases with no established treatment. Evaluating the effectiveness of transplantation can lay the foundation for the development of new treatments for these diseases, including retinitis pigmentosa with RPE-related gene abnormalities (such as RPE65, RDH5, and MERTK) [13,44,45,46,47,48,49,50,51], AMD with RPE atrophy [52,53,54,55,56]. There is currently no established treatment for RPE impairment, manifesting as severe myopia, retinal pigment epithelium, angioid streaks, Best disease, and Stargardt disease. On the other hand, for retinitis pigmentosa associated with the protein deletion type pathology caused by RPE65 gene abnormalities, gene therapy supplementing the wild-type gene was approved by the FDA in 2017, and its effectiveness has been already reported [47]. Meanwhile, a clinical trial protocol for the gene therapy of the RPE65 gene abnormality in *MERTK* patients failed to achieve the efficacy results of *RPE65* trials [13].

However, since gene therapy is effective only in the early stages of photoreceptor degeneration [57], it is difficult to apply in cases with advanced stages. Therefore, its availability for RPE impairment is quite limited, and there is still no cure for RPE degeneration and loss. These observations suggest that regenerative medicine by cell transplantation might be needed for the late stages of these diseases in the future.

A development framework will be established to help contribute to the growth of regenerative medicine and other multifaceted and comprehensive treatment strategies. Through this framework, we will be able to establish diagnostic criteria for a new disease concept called “RPE impaired disease” and set standard endpoints modeled on RPE transplantation to develop new treatments for various retinopathy with RPE impairment, for which no cure has been established.

### 4.2. Examination of Appropriate Efficacy Evaluation Method

**Figure 4:**
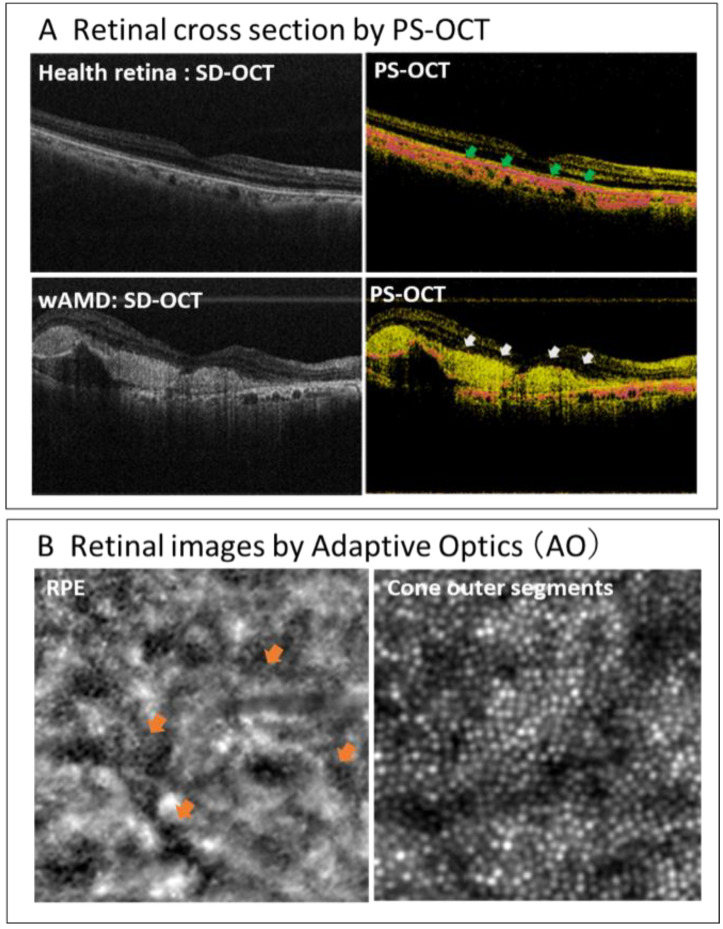
New in vivo diagnostic imaging tools for RPE cell visualization.

It is necessary to develop an evaluation method that can objectively evaluate cell transplantation itself, instead of the conventional efficacy evaluation item focusing on visual acuity, which may be subjective. In previous clinical studies of RPE cell transplantation, the number of transplanted cells and the transplantation site was limited, thus, significant visual acuity and visual field improvements could not be expected. Therefore, it is important to evaluate the anatomical recovery of the retinal structure at the transplant site [6,9,12]. However, it is difficult to observe the transplanted cells and their surrounding tissues in detail with the current ophthalmic diagnostic equipment. To address these problems, we introduce the polarized OCT and adaptive optics as novel in vivo diagnostic imaging tools for RPE cell visualization (Figure 4). Since the polarized OCT can visualize the melanin pigment abundant in healthy RPE cells, it is possible to distinguish between RPE abnormal sites and normal sites [58]. In addition, adaptive optics enables retinal observation at the cellular level (Figure 4). A more precise evaluation will be possible in the future if sample cells are engrafted at the RPE abnormal site and the normalization of the retinal structure is visualized using these techniques.

Visualization of the RPE layer using polarized optical coherence tomography (PS-OCT) (A). The RPE layer is clearer in the healthy retina (green arrows in the upper right panel) and wAMD retina (white arrows in the lower right panel) compared to those in SD-OCT images (left side panels). Retinal images obtained using adaptive optics (AO) (B). RPE cells with a hexagonal shape are observed in the retina with sub-retinal fluid (orange arrows in the left panel), and the outer segments of the cones are observed in the healthy retina (right panel).

### 4.3. Optimization of Cell Resources

For the practical application of retinal regenerative medicine, it is important to establish a comprehensive strategy from manufacturing to therapeutic methods. For example, cost reduction is expected by promptly reviewing the cell sources and manufacturing processes. While autologous transplantation is the best treatment, it is expected to be costly. HLA-matched allogeneic transplantation without immune suppression will be safer for the elderly, but the preparation of iPS cells with various types of HLA incur high costs. On the other hand, if the cells have partially-deleted HLA, only one cell line will be sufficient to cover all patients. The Center for iPS Cell Research and Application offers iPS cells that partially disrupt HLA. Since these cells are less likely to be rejected, even by recipients with different HLA haplotypes, there is a possibility of reducing the cost of raw cell materials if they are put into practical use. In fact, in the clinical research on pluripotent cell-derived RPE cell transplantation conducted so far, HLA-compatible allogeneic transplantation, which can be performed without the use of immunosuppressive drugs, was carried out for the elderly. However, withdrawal cases due to the side effects of immunosuppressive drug administration have also been reported [5,10,11]. From that experience, it may be challenging to enroll HLA-matched patients and administer immunosuppressants to the elderly and those with RPE-impaired disease with rare retinal degenerative diseases. As such, future efforts should be developed to counter these.

### 4.4. Rise of New Technology for Manufacturing

**Figure 5:**
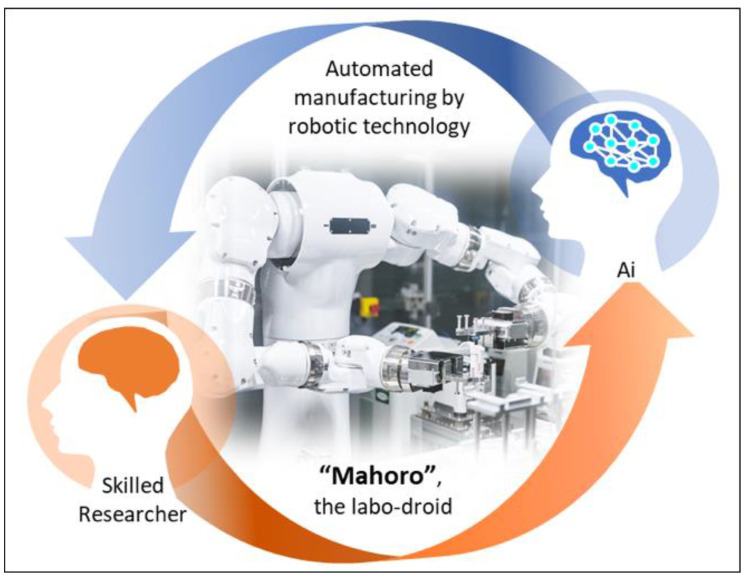
Automated manufacturing by robotic technology.

In light of the advancements in regenerative medicine, the application of robot technology for purposed from the evaluation of differentiation conditions to the actual manufacturing (Figure 5) can lead to new non-invasive cell quality evaluation methods. Furthermore, the sophistication of networks through the development of the Internet of Things and the analysis of big data obtained from clinical studies by artificial intelligence will evolve into entirely new medical treatments.

“MAHORO,” the labor droid, can support researchers in preparing automated manufacturing systems for cell products of regenerative medicines using various equipment. This is achieved by transferring techniques established by a skilled researcher and the autonomous control driven by artificial intelligence (Ai) to optimize the manufacturing conditions.

### 4.5. Regarding Regulatory Issues Related to Regenerative Medicine

As described previously, regenerative medicine is producing remarkable progress partially due to the new regulation, but the reaction in Europe and the United States to Japanese regenerative medicine-related laws (created in 2014) is not always favorable. The British scientific journal Nature published an article stating that randomized controlled trials (RCTs), a verification method for scientifically evaluating the therapeutic effect by comparing patients in the treated group and the non-treated group, have not been implemented. Furthermore, they advocated the introduction of a more transparent system. The purposes of these laws will indeed be defeated if people utilize these techniques incorrectly. However, regenerative medicine is still an emerging field, and the cells will behave differently in each individual according to the microenvironment. It is difficult at the beginning to prepare enough cells for an RCT to show statistically significant results. In addition, due to the rapid pace of progress in stem cell science, therapies may be outdated by the time large RCTs are finished. We do not know yet whether the conventional regulation for small-molecule drugs is appropriate.

On the other hand, in March, the Japan Society for Regenerative Medicine believes that a new approach is needed to deliver treatment to patients with diseases with no approved therapeutic drug as soon as possible. For rare diseases, RCT is not essential for all products (https://en.jsrm.jp/news/news-162/ accessed on 20 April 2021). Behind this growing debate is international competition, such as in expediting the establishment of examinations and approvals under the regenerative medicine advanced treatment designation system based on the 21st Century Medical Law enacted in 2016 in the United States. However, the important point is that we should always first consider the benefits to the patients. Furthermore, we should not only focus on the cell-related risks, the main concern of regulation, but also examine the risks of treatment, such as immune suppression or surgery, and the “risks of inaction.”

## 5. Conclusions

In the future, it is expected that efforts aimed at the practical application of stem cells to AMD will also contribute to various related fields. The superiority of retinal regenerative medicine, that is, the reasonable manufacturing cost due to the small number of cells used, the established surgical equipment and techniques, and the highly accurate diagnostic imaging equipment that enables in vivo direct observation is maximized. Hopefully, ophthalmologists will take the lead in designing retinal regenerative medicine strategies for patients suffering from incurable retinal degenerative diseases.

## Figures and Tables

**Table 1:** Stem cell therapies for AMD with pluripotent stem cell derived-RPE.

No.	Study Title	Sponsor/Collaborators	Intervention	Age	Phases	No. of Subjects	Start/Completion Date	Status	Study ID
1	A Study of transplantation of autologous induced pluripotent stem cell (iPSC) derived retinal pigment epithelium (RPE) cell sheet in subjects with exudative age-related macular degeneration	the Laboratory for Retinal Regeneration, RIKEN Center for Developmental Biology	autologous hiPSC derived RPE cell sheet	50 years and older	P1	1	October 2013 /September 2018	completed	UMIN000011929
2	Autologous Transplantation of Induced Pluripotent Stem Cell-Derived Retinal Pigment Epithelium for Geographic Atrophy Associated With Age-Related Macular Degeneration	National Institutes of Health Clinical Center, Bethesda, Maryland, U.S.	Combination Product: hiPSC-derived RPE/PLGA scaffold	55 years and older	P1	20	July 2020 /March 2029	Recruiting	NCT04339764
3	A Study Of Implantation Of Retinal Pigment Epithelium In Subjects With Acute Wet Age Related Macular Degeneration	Moorfields Eye Hospital NHS Foundation Trust, London, U.K.	PF-05206388: RPE living tissue equivalent for intraocular use in the form of a monolayer of RPE cells immobilized on a polyester membrane.	60 years and older	P1	2	July 2020 /March 2029	Recruiting	NCT04339764
4	Study of Subretinal Implantation of Human Embryonic Stem Cell-Derived RPE Cells in Advanced Dry AMD	Retinal Arizona LTD, Phoenix, Arizona, U.S./Retina-Vitreous Associates Medical Group, Beverly Hills, California, U.S. and others	CPCB-RPE1 (Human Embryonic Stem Cell-Derived RPE Cells Seeded on a Polymeric Substrate)	55 years to 85 years	P1/2a	16	July 2019 /June 2023	Active, not recruiting	NCT02590692
5	A Study of transplantation of allogenic induced pluripotent stem cell (iPSC) derived retinal pigment epithelium (RPE) cell suspension in subjects with neovascular age related macular degeneration	the Laboratory for Retinal Regeneration, RIKEN Center for Developmental Biology, Kobe, Japan/ Kobe City Medical Center General Hosital, Kobe, Japan	Subretinal transplantation of allogenic hiPSC derived RPE cells	50 years to 85 years	P1	5	February 2017 /October 2021	Active, not recruiting	UMIN000026003
6	Stem Cell Therapy for Outer Retinal Degenerations	Federal University of Sao Paulo, Sao Paulo, Brazil	injection of hESC derived RPE in suspension/Procedure: injection hESC derived RPE seeded in a substrate	18 years to 90 years	P1/2	15	September 2016 /July 2020	Completed	NCT02903576
7	Subretinal Transplantation of Retinal Pigment Epitheliums in Treatment of Age-related Macular Degeneration Diseases	Chinese Academy of Sciences/Beijing Tongren Hospital, China	hESC derived RPE	55 years and older	P1/2	10	January 2018 /December 2020	Recruiting	NCT02755428
8	Safety and Efficacy of Subretinal Transplantation of Clinical Human Embryonic Stem Cell Derived Retinal Pigment Epitheliums in Treatment of Retinitis Pigmentosa	Qi Zhou, Chinese Academy of Sciences	hESC derived RPE	18 years and older	P1	10	May 2020 /December 2021	Recruiting	NCT03944239
9	Treatment of Dry Age Related Macular Degeneration Disease With Retinal Pigment Epithelium Derived From Human Embryonic Stem Cells	Chinese Academy of Sciences/ The First Affiliated Hospital of Zhengzhou University, China	hESC derived RPE	55 years and older	P1/2	15	September 2017 /December 2020	Recruiting	NCT03046407
10	Safety and Tolerability of Sub-retinal Transplantation of Human Embryonic Stem Cell Derived Retinal Pigmented Epithelial (hESC-RPE) Cells in Patients With Stargardt’s Macular Dystrophy (SMD)	Astellas Institute for Regenerative Medicine/Astellas Pharma Inc., U.S.	hESC derived RPE (MA09-hRPE)	18 years and older	P1/2	15	November 2011 /September 2015	completed	NCT01469832
11	A Follow up Study to Determine the Safety and Tolerability of Sub-retinal Transplantation of Human Embryonic Stem Cell Derived Retinal Pigmented Epithelial (hESC-RPE) Cells in Patients With Stargardt’s Macular Dystrophy (SMD)	Astellas Institute for Regenerative Medicine/Astellas Pharma Inc., U.S.	hESC derived RPE (MA09-hRPE)	18 years and older		12	January 2013 /October 2019	completed	NCT02941991
12	Sub-retinal Transplantation of hESC Derived RPE(MA09-hRPE) Cells in Patients With Stargardt’s Macular Dystrophy	Astellas Institute for Regenerative Medicine/Astellas Pharma Inc., U.S.	hESC derived RPE (MA09-hRPE)	18 years and older	P1/2	13	April 2011 /August 2015	completed	NCT01345006
13	Safety and Tolerability of Sub-retinal Transplantation of hESC Derived RPE (MA09-hRPE) Cells in Patients With Advanced Dry Age Related Macular Degeneration	Astellas Institute for Regenerative Medicine/Astellas Pharma Inc., U.S.	hESC derived RPE (MA09-hRPE)	55 years and older	P1/2	13	April 2011 /August 2015	completed	NCT01344993
14	Long Term Follow Up of Sub-retinal Transplantation of hESC Derived RPE Cells in Stargardt Macular Dystrophy Patients	Astellas Institute for Regenerative Medicine/Astellas Pharma Inc., U.S..	hESC derived RPE (MA09-hRPE)	18 years and older	P1	13	July 2012 /June 2019	completed	NCT02445612
15	Long Term Follow Up of Sub-retinal Transplantation of hESC Derived RPE Cells in Patients With AMD	Astellas Institute for Regenerative Medicine/Astellas Pharma Inc., U.S.	hESC derived RPE (MA09-hRPE)	18 years and older		11	February 2013 /August 2019	completed	NCT02463344
16	A Phase I/IIa, Open-Label, Single-Center, Prospective Study to Determine the Safety and Tolerability of Sub-retinal Transplantation of Human ES Cell Derived RPE (MA09-hRPE) Cells in Patients With Advanced Dry Age-related Macular Degeneration (AMD)	Astellas Institute for Regenerative Medicine/Astellas Pharma Inc., U.S.	hESC derived RPE (MA09-hRPE)	55 years and older	P1/2a	12	September 2012 /June 2020	Active, not recruiting	NCT01674829
17	A Safety Surveillance Study in Subjects With Macular Degenerative Disease Treated With Human Embryonic Stem Cell-derived Retinal Pigment Epithelial Cell Therapy	Astellas Institute for Regenerative Medicine/Astellas Pharma Inc., U.S.	hESC derived RPE (MA09-hRPE)	18 years and older	P1/2	36	January 2018 /December 2029	Enrolling by invitation	NCT03167203
18	Retinal Pigment Epithelium Safety Study For Patients In B4711001	Moorfields Eye Hospital NHS Foundation Trust, U.K.	hESC derived RPE	60 years and older		2	September 2016 /October 2020	Active, not recruiting	NCT03102138
19	Safety and Efficacy Study of OpRegen for Treatment of Advanced Dry-Form Age-Related Macular Degeneration	Lineage Cell Therapeutics, Inc./CellCure Neurosciences Ltd., Israel	OpRegen	50 years and older	P1/2	24	August 2015 /December 2024	Recruiting	NCT02286089

**Table 2:** Stem cell therapies for AMD with somatic stem cells.

No.	Study Title	Sponsor/Collaborators	Intervention	Age	Phases	No. of Subjects	Start/Completion Date	Status	Study ID
1	Study of Human Central Nervous System Stem Cells (HuCNS-SC) in Age-Related Macular Degeneration (AMD)	Retina-Vitreaous Associates Medical Group, Los Angeles, California, United States/ Byers Eye Institute at Stanford, Stanford Hospital and Clinics, Palo Alto, California, United States/ New York Eye and Ear Infirmary, New York, United States and others	hCNS-SC cells	50 years and older	P1/2	15	June 2012 /June 2015	completed	NCT01632527
2	Long-Term Follow-up Safety Study of Human Central Nervous System Stem Cells in Subjects With Geographic Atrophy of Age-Related Macular Degeneration	Retina Foundation of the Southwest, Dallas, Texas, United States	hCNS-SC cells	50 years and older	P1	8	April 2014 /May 2016	Terminated	NCT02137915
3	Intravitreal Bone Marrow-Derived Stem Cells in Patients With Macular Degeneration	Rubens Siqueira Research Center, Sao Jose do Rio Preto, SP, Brazil	intravitreal injection of autologous bone marrow stem cell	18 to 80 years old	P1	20	August 2011 /September 2013	Completed	NCT01518127
4	Stem Cells Therapy in Degenerative Diseases of the Retina	Department of Ophthalmology, Szczecin, Poland	autologous bone marrow-isolated stem/ progenitor cells transplantation	18 Years to 65 Years	P1	30	December 2018 /February 2020	Enrolling by invitation	NCT03772938
5	Stem Cell Ophthalmology Treatment Study II	MD Stem Cells, Westport, Connecticut, United States/ MD Stem Cells, Coral Springs, Florida, United States/ Fakeeh University Hospital, Dubai, United Arab Emirates	autologous bone marrow derived stem cells	18 Years and older	NA	500	January 2016 /January 2022	Recruiting	NCT03011541
6	Clinical Trial of Autologous Intravitreal Bone-marrow CD34+ Stem Cells for Retinopathy	University of California Davis, Sacramento, California, United States	CD34+ bone marrow stem cells intravitreal	18 years and older	P1	15	July 2012 /January 2022	Enrolling by invitation	NCT01736059
7	Stem Cell Ophthalmology Treatment Study	MD Stem Cells, Westport, Connecticut, United States	autologous bone marrow derived stem cells	18 years and older	P1	300	August 2012 /July 2020	Enrolling by invitation	NCT01920867
8	Study of Assess the safety and effects of cells injected in Dry Macular Degeneration	Bioheart Sunrise, Florida, United States	adipose derived stem dells	50 years to 90 years	NA	0	December 2013 /December 2016	Withdrawn	NCT02024269

NA: not available.

## Data Availability

Data available in publicly accessible repository that does not issue DOIs.
